# A Comprehensive Review on Digital Detox: A Newer Health and Wellness Trend in the Current Era

**DOI:** 10.7759/cureus.58719

**Published:** 2024-04-22

**Authors:** Gaurang Anandpara, Ashish Kharadi, Prakash Vidja, Yashkumar Chauhan, Swati Mahajan, Jitendra Patel

**Affiliations:** 1 Biochemistry, Chimanlal Ujamshibhai Shah Medical College & Hospital, Surendranagar, IND; 2 Surgery, Gujarat Medical Education and Research Society Medical College, Godhra, IND; 3 Pathology, Pre Cure Pathology Laboratory, Morbi, IND; 4 Medicine, Smt. Nathiba Hargovandas Lakhmichand Municipal Medical College, Ahmedabad, IND; 5 Physiology, Gujarat Medical Education and Research Society Medical College, Godhra, IND; 6 Physiology, Gujarat Medical Education and Research Society Medical College, Vadnagar, IND

**Keywords:** digital detox, wellbeing, psychological barriers, technology, behaviour

## Abstract

This research investigates the effects of an electronic detox treatment on the utilization of social media and smartphones, addiction levels, and the general health of individuals. Remarkably, individuals discovered that the digital detox was less challenging than anticipated, with a significant number expressing sensations of pleasure and alleviation. Although a few individuals encountered instances of alienation and solitude, the majority managed to adapt to the limited availability of the internet. Notably, individuals saw heightened tedium and replaced their use of social networking sites with additional tasks using screens. After the procedure, measures demonstrated favorable or neutral enhancements in addictions and health-related results. The quantitative findings indicate an increased understanding of online conduct and the use of self-regulating strategies. Concrete recommendations put forward by respondents include reducing stringent deadlines, implementing personalized limitations, and devising tactics to regulate alerts and their use. These observations may be used to shape subsequent digital detox programs in order to improve their efficacy and increase participation from participants.

## Introduction and background

Digital detox means a person or individual stays disconnected from devices or social media for a defined duration. According to research, not using social media can have a significant impact on a person's health and well-being. There is a clear difference in approach between daily life and holiday encounters, marked by a desire for a new rhythm and a pause from the typical schedule [1]. Furthermore, elements such as the pursuit of unique encounters and the freedom from everyday responsibilities play a significant role in this transformation [2]. While on holiday, people often have mental barriers when it comes to accessing the web [3] and meet difficulties with technology [4], resulting in changes in their behaviour compared to when they are at home. These disparities are influenced and driven by the frequency of using electronic devices [3,5]. Past research has examined chiefly the unequal access to digital resources in the tourism industry, particularly among visitors from technologically advanced nations [6,7]. However, the widespread usage of cell phones has reduced these discrepancies [8,9], resulting in less incentive to detach oneself from digital connection [1,5,10]. Concerns about the effect of excessive information and multitasking on well-being are a result of the lack of research on health inequality.

This review paper explores the correlation between tourism and quality of life, well-being, and health, specifically focusing on the digital gap between home and holiday destinations. This gap involves disparities not just in physical entry, utilization, and abilities but also in intention, drive, sentiment, inherent variables, and subjectivity. Experts have redirected their attention from obstacles associated with infrastructure and technology to hindrances linked to skills and utilization [3]. In addition, new challenges have arisen, such as a lack of trust [6], the need for leisure and personal growth, and other psychological, inherent, and emotional limitations. Although economic, technical, and socio-capability frameworks have been used to examine the previous obstacles [6], the latter have yet to be given as much focus. It is necessary to have a more comprehensive theoretical structure that includes mental and health-oriented methods. Motivations for travelling encompass a wide range of reasons, including the desire to unwind, excitement, keeping connections, improving self-esteem, and encouraging development in oneself. This highlights the significance of examining not only exterior and goal barriers but also inherent and personal inspiring challenges that are linked to digital disparities, such as the need for escape, stress relief, and well-being. This study examines various hypotheses that aim to clarify the digital divide between home and vacation destinations and examines the current barriers to accessing the internet while on vacation [11].

## Review

Methodology

**Figure 1:**
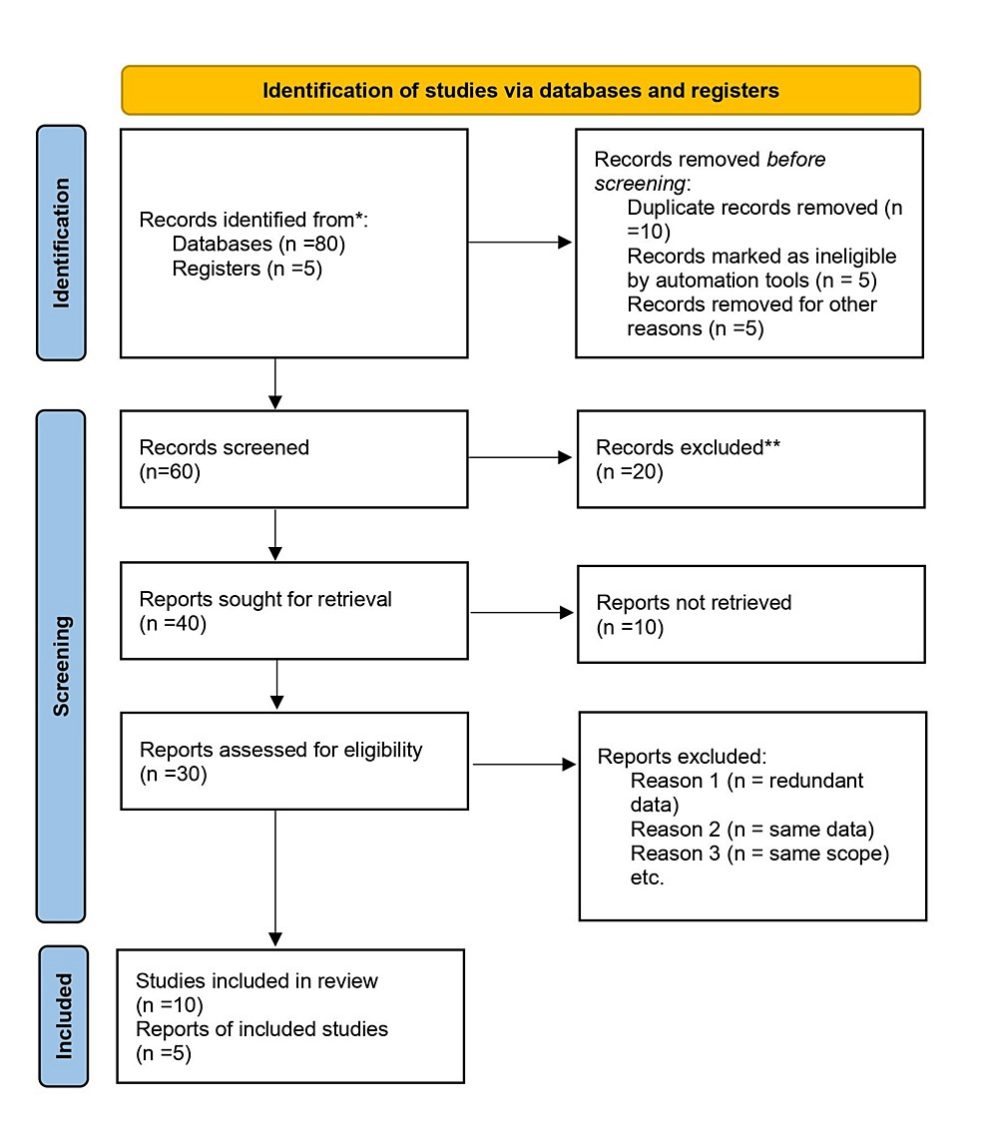
PRISMA flow chart PRISMA: Preferred Reporting Items for Systematic Reviews and Meta-Analyses *Number of records identified from databases: PubMed (n=10), Web of Science (n=15), Google Scholar (n=55), Registers (publications or conference proceedings) (n=5)

Below is a comprehensive overview of the resources and techniques that were used for this review paper on digital detox. The study was initiated by finding relevant databases and registries to conduct searches using the keywords "digital detox", "social media addiction", "smartphone addiction", "health and wellness" and "behavioural changes". These databases included platforms such as PubMed, Web of Science, and several more. Additionally, it was important to take into account grey sources of literature and manually search through important publications or conference proceedings. Following the search, any duplicate entries were eliminated and the abstracts and titles of the remaining papers were evaluated to determine their relevance. To complete this phase, it was necessary to establish precise criteria for what should be included and excluded in relation to the subject. The whole content of the possibly applicable studies was examined to verify whether they met the criteria for inclusion. Once again, the outcome was determined by the parameters that were already established. The conclusive collection of papers that satisfied all criteria was selected for inclusion in the systematic review. The PRISMA (Preferred Reporting Items for Systematic Reviews and Meta-Analyses) flow diagram illustrated the progression of information across several stages of this review. The data visualization displayed the quantity of recognized records, both retained and omitted, together with the specific reasons for exclusions (Figure 1).

Inclusion Criterion

This review looks at studies written in English that explore digital detox programs where people choose to limit their digital device use. The studies focus on abstaining from devices like smartphones or specific types of use, such as social networking. They use statistics to compare the effects of these programs with a control group or baseline. The review covers a wide range of outcomes, including health, social relationships, and cognitive performance.

Exclusion Criterion

It excludes studies that only look at internet gaming disorders or addiction, not smartphone use, and doesn't include qualitative studies.

Literature review

The practice of digital detox, which refers to the deliberate abstention from electronic devices such as smartphones, is gaining popularity in the health and wellness industry. It is considered a viable solution to mitigate the negative consequences of excessive smartphone usage on one's well-being, social connections, and other areas of life. Radtke et al. performed a comprehensive analysis of existing research to evaluate the efficacy of digital detox programmes in enhancing factors such as well-being and health, social connections, discipline, or productivity [12]. Their comprehensive analysis, which included 21 trials with a total of 3,625 people, revealed diverse outcomes seen in different investigations. Several studies have shown favourable outcomes resulting from interventions, whereas others have shown little impact or even adverse impacts on well-being [12]. A further study conducted by Coyne and Woodruff examined the consequences of a 14-day period in which young individuals abstained from social media, limiting their use to 30 minutes each day [13]. According to a study, this detox has been shown to increase addiction to smartphones and social media while also improving sleep quality, overall life satisfaction, stress levels, perceived healthiness, and supportive connections [13]. However, it is essential to acknowledge that the efficacy of digital detoxes may significantly differ depending on the person and the precise parameters of the detoxification process. Hence, it is crucial to do more study in order to fully understand the processes of change and to create compelling digital detox strategies [12]. To summarize, while digital detox has the potential to reduce the adverse consequences of excess digital device use, its efficacy might be inconsistent, highlighting the need for more investigation to enhance these therapies [12-13]. Díaz-Meneses and Estupinán-Ojeda investigated the tangible and measurable obstacles that contribute to the digital divide between staying in residence and going on vacation, as well as the inherent and personal factors that hinder staying connected online when at a location [11]. Their research, which went beyond conventional economic and technical reasons to include aspects related to well-being and psychology, highlighted the importance of "detox" incentives in explaining the variations in digital behaviour between home and vacation places [11] (Table 1). A *Times of India* article explores the impact technological advances have on human behaviour and the significant transformations brought about by digital connections [14]. This highlights the risks associated with extended periods of using screens and emphasizes the need to have a well-defined strategy for taking breaks from digital devices [14]. The review proposes the establishment of areas devoid of technological devices and the intentional use of information to achieve a well-rounded digital existence. In general, the evidence suggests that digital detox may be a valuable approach for addressing the adverse effects of excessive usage of digital devices. However, its efficacy could be more consistent and additional study is needed to enhance treatments [13,14].

**Table 1:** Study design for the digital detox experiment under consideration [12, 15-33]

No	Design	Findings	Author(s)
1	Synthesis of existing evidence	Provides a synthesis of existing evidence of associations between digital detox and different outcomes	Radtke R, et al. [12]
2	Phenomenological exploration	Explores self-tracking in the context of digitization	Kent R [15]
3	Exploratory study	Explores the role of digital detox applications	Schmuck D [16]
4	Qualitative study	Examines media resistance and the promise of authenticity	Syvertsen T & Enli G [17]
5	Phenomenological exploration	Explores cultivating digital mindfulness	Stanovsek SK [18]
6	Conceptual analysis	Explores addiction to digital devices during the holidays	Emek M [19]
7	Perception study	Examines perceptions of social media users of digital detox apps	Nguyen VT [20]
8	Literature review	Discusses inappropriate smartphone use as an emerging public health problem	van Velthoven MH, et al. [21]
9	Correlational study	Relates physiological stress measurements to smartphone usage	Anrijs S, et al. [22]
10	Conceptual analysis	Discusses digital detox as a strategic tool for tourism development	Dimova D [23]
11	Qualitative analysis	Captures contested and conflicting insights on digital well-being and detoxing	Mutsvairo B & Ragnedda M [24]
12	Book	Discusses breaking free from wellness traps	Harrison C [25]
13	Anthropological study	Provides an anthropological view of digital harm and addiction	Sutton T [26]
14	Cross-sectional study	Identifies predictors of liking health and fitness-related content on social media	Carrotte ER, et al. [27]
15	Book	Discusses global consumer trends for 2015	Kasriel-Alexander D [28]
16	Discourse analysis	Analyzes media representation of digital-free tourism	Li J, et al. [29]
17	Mapping study	Maps new roles and responsibilities in digital well-being in tourism	Stankov U & Gretzel U [30]
18	Article	Explores digital dieting as a response to information obesity	Brabazon T [31]
19	Review article	Discusses opportunities and challenges for contactless healthcare services	Lee SM & Lee DH [32]
20	Report	Discusses healthcare transformation in the era of digital health	Bhavnani SP, et al. [33]

The study by Mirbabaie et al. thoroughly examines the widespread impact of technological advances (IT) on society as a whole, namely in the domain of labour and its limitations [34]. Professionals, particularly knowledge workers, constantly have access to electronic gadgets during their hours at work, while social networking sites and electronic pastimes dominate a significant portion of people's free time. The prolonged use of screens may have substantial consequences on persons' welfare, resulting in technological anxiety, which is a detrimental effect generated in either a direct or indirect way by computers. The COVID-19 epidemic has worsened this problem by blurring the boundaries between work and home life. A study referenced in the evaluation revealed that 86 per cent of those surveyed held the belief that the failure to detach from technological gadgets outside of working hours had an adverse impact on their overall state of well-being. The notion of "digital detox" is gaining popularity as a means to combat technological anxiety and its effects on both happiness and performance. Engaging in a digital detox entails regularly withdrawing from information technology and using techniques to decrease one's involvement with it.

Nevertheless, the understanding and practical examination of the electronic detox still needs more clarity. A preliminary study indicates inconclusive findings on its efficacy in enhancing personal well-being. The review highlights the significance of digital detoxification in mitigating the adverse effects of the over-utilization of digital devices. Nevertheless, it underscores the need for more investigation to comprehend its processes and devise efficacious therapies [35] (Table 2).

**Table 2:** Assessment activities in digital detox [35]

Scale	Description
Time spent online	Measures the amount of time spent online.
Internet preoccupation	Assesses constant thinking about previous online activity or anticipation of the following online session.
Efforts to curtail internet use	Evaluates repeated unsuccessful efforts to control, cut back, or stop internet use.
Mood regulation via the internet	Measures the use of the internet as a way of escaping from problems or relieving a dysphoric mood.
Risking job or relationship	Assesses the risk of losing a significant relationship, job, educational or career opportunity because of the internet.
Lying about time spent online	Evaluates lying to family members, therapists, or others to conceal the extent of involvement with the internet.

Respondents in the research, who were assessed using either device-based or reported methods to quantify their cell phone and internet use, showed noteworthy decreases in their usage throughout the duration of the study. Notably, a brief two-week evaluation showed a possible rebounding operation, as consumption patterns reverted to levels seen before the treatment began, suggesting a propensity for relapse. The digital detox resulted in a reduction in both smartphone and internet dependency, which continued to be seen during the two weeks afterwards, in comparison to the initial levels [36]. The results are consistent with other studies [37], which also documented decreases in dependence on smartphones when use is restricted. Furthermore, the research indicates that abstaining from social media use on smartphones might decrease both the inclination towards smartphone dependence and the use of social media. These effects have been demonstrated for a significant period, lasting several weeks after the treatment [13,37] (Table 3).

**Table 3:** Some permissible activities in the situation of digital detox [37-38]

Activity	Description
Unplugging	Disconnect from all digital devices for a certain period.
Time management	Set specific times for using and not using digital devices.
Mindful usage	Be aware of the time and purpose of using digital devices.
Physical activity	Engage in physical activities like walking, yoga, etc., to distract from digital devices.
Reading books	Spend time reading physical books instead of e-books.
Nature time	Spend time outdoors, in nature, away from digital devices.

At first, the writers expected challenges in enrolling volunteers for the detox research, believing that many would see the detox as onerous or impractical. Surprisingly, both the study team and volunteers discovered that a significant number of persons derived pleasure and experienced a sense of relief from the detox process, believing it to be less challenging than anticipated. Although several individuals experienced sensations of alienation and loneliness, these adverse emotions did not result in the cessation of their involvement. The majority of those surveyed had difficulty but were able to handle the 30-minute restriction on the internet, showcasing their capacity to adjust their daily habits to accept limited usage of the internet. A significant number of individuals reported heightened feelings of ennui throughout the intervention phase, notably during brief intervals between tasks. This discovery aligns with other research indicating that diminishing one's engagement with social media will lead to heightened feelings of ennui. Respondents also observed substituting social networking sites with different kinds of Time spent on screens, perhaps without being aware of it, as a result of the regular aspect of technological use, especially for recreational purposes. The research addresses a hitherto unexplored aspect by including a further assessment two weeks following the detoxification process. Qualitative reports revealed that some individuals promptly engaged in online activities after the treatment, although statistical analysis demonstrated favourable or neutral enhancements in addictions and related to health results. Respondents furthermore developed a heightened awareness of their online communication patterns and implemented measures to control their use. For future detoxes, the writers propose implementing time constraints that are a bit less stringent, tailoring these constraints to each participant's usage habits, reducing the number of social media alerts, carefully limiting accessibility to specific uses, and urging subjects to delete or log out of their social networking accounts throughout the detox period. Applying these observations might improve the efficiency and involvement of participants in future detoxification programmes [13,38] (Table 3).

In his 2022 review, Nguyen VT explores how users perceive digital detox programmes and how these impressions are influenced by their personalities and technology-related factors [20]. The research used a survey methodology and using Generalized Structured Component Analysis (GSCA). It included a sample of 263 users who utilize detox applications to mitigate the impact of online disruptions. The results indicated that there was a strong relationship between the intention to engage in a behaviour and the actual behaviour shown. Achievement expectation, expectation of effort, and social context had a favourable impact on behavioural intention [20] (Table 1).

Furthermore, agreeableness and extroversion had a favourable effect on achievement standards, but extroversion impacted work expectations. Neuroticism was shown to have a negative correlation with the expectation of work required to use internet-based detox applications. Nevertheless, the presence of conducive circumstances did not forecast the inclination to engage in a particular behaviour. Similarly, the willingness to explore new experiences was independent of the anticipated level of achievement. Furthermore, the trait of dedication was not shown to be associated with the perceived level of effort required. In summary, the suggested model accounted for 56.68% of the variability, indicating that teachers, politicians, and software developers should take individual factors into account when developing intervention strategies to address educational challenges associated with online distractions. Although the study offers helpful information, further research is required to comprehensively comprehend these linkages and create operational digital detox strategies [39].

In their 2015 work, Ugur and Koca examine the difficulties presented by cell phones in educational institutions [39]. The research conducted a poll among students from various divisions to assess the degree to which portable technology is seen as a significant disruption in lectures and during tests. The researchers investigated the social dimensions of technology connectedness, including phenomena such as phubbing (disregarding someone in favour of a cell phone), academic dishonesty, and student perspectives on cell phone policies and teacher conduct. The key results reveal that a substantial proportion of learners use their cell phones for private purposes while attending classes. The dimensions of the learning environment have a considerable influence on student conduct since more prominent educational institutions tend to result in more unrestrained behaviour, such as phubbing. A significant number of students confess to being easily diverted by their peers and accept the possibility of engaging in academic dishonesty with the use of their mobile devices. The research highlights the need for a digital detox, particularly in academic environments, in order to promote efficient learning and uphold integrity in education. It indicates that the inappropriate use of mobile devices in educational settings is a significant problem that demands the focus of teachers and regulators [39].

The research conducted by Umasankar M et al. (2022) investigates the effects of digital detoxification on the welfare of employees, emphasizing the difficulties encountered in attaining happiness in an electronic work environment [40]. The authors contend that during the current era of digitalization, professionals in non-manual occupations greatly depend on electronic devices, rendering it challenging to envision a work routine that is entirely free from technological tools. With the growing prevalence of remote work, the welfare of employees has emerged as a significant concern. The study used a design based on descriptive statistics and applied a survey approach to investigate employee digital detoxification and digital wellness. An organized questionnaire was used to examine different facets of these phenomena. The survey specifically targeted professionals in the IT industry who have enthusiastically adopted working from home. The results, examined using Structural Equation Modelling, may deviate from previous research and provide fresh perspectives for professionals in the management of human resources. The study highlights the value of digital detoxification in preserving employee welfare, particularly in the setting of remote employment, proposing it as a crucial tactic for organizations to improve staff efficiency and effectiveness [40].

The research conducted by Handa and Ahuja (2020) examines the prevalence of addiction to smartphones among young individuals in India [41]. The findings indicate that almost 25% of the participants had elevated scores on the cell phone dependency measure. The poll, targeting young adults between the ages of 18 and 25, revealed that the majority of respondents allocated a significant portion of their time to using applications such as WhatsApp and engaging with social media sites. The study found the fear of losing out as a factor that might predict problematic smartphone use. It observed that those who use their smartphones excessively tend to have worse overall sleep. The writers highlighted the significance of electronic detox in reducing the excessive use of smartphones and minimizing its adverse effects. They provided significant insights into the prevalence and repercussions of addiction to smartphones among young people in India [41].

Wilcockson et al. (2019) conducted a study to examine the impact of 24 hours without smartphone use on mood, anxiety, and desire. The results revealed that only craving was influenced by the duration of abstinence. The research indicates that excessive use of smartphones may not fulfil the requirements since signs often linked with dependence on drugs were not regularly seen. The results of this study add to the ongoing discussion on how to define and understand behavioural addictions. Additionally, these findings may have significant consequences for resolving issues related to excessive smartphone usage [42].

Vialle et al. (2023) explore the perspectives of those who engage in digital detoxification. The research was conducted in semi-structured interviews with a total of five women and two men who had experienced digital detoxes, resulting in the identification of two themes. "Resisting temptation: the digital diet" captures the challenge that people have when trying to restrict their use of digital devices. "In search of the perfect self: actual versus ideal" captures the underlying drive for digital detoxes, which arise from a perceived discrepancy between a person's desired self-image and their online persona. The study demonstrates that technological innovation, particularly cell phones and social networking sites, is seen as a menace to one's principles, self-control, and self-worth. Although digital detoxing is considered a method to decrease this pride, several individuals nevertheless see digital tools as essential for handling job and social interactions. The authors propose that individuals facing self-discrepancy might potentially derive advantages from being exposed to scholarship that emphasizes the positive aspects of digital technology. Such exposure may lead to a change in their perspectives, shifting their focus towards moderate instead of complete abstention. This research offers distinctive perspectives into the motives and observations of persons who engage in digital detoxes, revealing the complex connection between the use of electronic devices and one's perception of oneself [43].

Hager et al. (2023) conducted a comprehensive analysis of approaches used in digital detox research, which was delivered at the eighteenth International Symposium on Wirtschaftsinformatik [44]. The contributors performed a comprehensive analysis of the existing literature, finding 65 pertinent investigations and classifying them according to their study strategy, methodology, and number of participants. The study identified five main study domains: interaction, instruction, travel, happiness and wellness, and job culture. The report offers analytical ideas to enhance the scientific study of digital detox, an area of study that is becoming more important but is still in its early stages. The text underscores the possible adverse effects of extensive reliance on technology on both happiness and efficiency while emphasizing the increasing inclination towards abstaining from online activities. This work establishes a basis for future investigations regarding digital detox, providing valuable perspectives on methodology and proposing potential avenues for additional study [44].

## Conclusions

This review article sheds light on the nuanced experiences of participants undergoing a digital detox intervention, highlighting both the challenges and benefits of reducing social media and smartphone usage. The findings suggest that while initial apprehensions about the detox were common, many participants found the experience manageable and even enjoyable. Notably, the detox led to positive changes in addiction and health-related outcomes, which were maintained post-intervention. The review also underscores the importance of personalized approaches to digital detox, as well as strategies to manage boredom and substitute screen time activities. These insights can inform the development of more effective and sustainable digital detox programs tailored to individual needs and preferences, ultimately promoting healthier digital habits and well-being.
